# Characterization and validation of an intra‐fraction motion management system for masked‐based radiosurgery

**DOI:** 10.1002/acm2.12573

**Published:** 2019-05-04

**Authors:** Nels C. Knutson, Brad J. Hawkins, Douglas Bollinger, S. Murty Goddu, James A. Kavanaugh, Lakshmi Santanam, Timothy J. Mitchell, Jacqueline E. Zoberi, Christina Tsien, Jiayi Huang, Clifford G. Robinson, Stephanie M. Perkins, Joshua L. Dowling, Michael R. Chicoine, Keith M. Rich, Gavin P. Dunn, Sasa Mutic

**Affiliations:** ^1^ Department of Radiation Oncology Washington University School of Medicine St. Louis MO USA; ^2^ Gateway Physics LLC St Louis MO USA; ^3^ Department of Neurosurgery Washington University School of Medicine St. Louis MO USA

**Keywords:** Gamma Knife, HDMM, Icon, IFMM, intra‐fraction motion, SRS

## Abstract

**Purpose:**

Characterize the intra‐fraction motion management (IFMM) system found on the Gamma Knife Icon (GKI), including spatial accuracy, latency, temporal performance, and overall effect on delivered dose.

**Methods:**

A phantom was constructed, consisting of a three‐axis translation mount, a remote motorized flipper, and a thermoplastic sphere surrounding a radiation detector. An infrared marker was placed on the translation mount secured to the flipper. The spatial accuracy of the IFMM was measured via the translation mount in all Cartesian planes. The detector was centered at the radiation focal point. A remote signal was used to move the marker out of the IFMM tolerance and pause the beam. A two‐channel electrometer was used to record the signals from the detector and the flipper when motion was signaled. These signals determined the latency and temporal performance of the GKI.

**Results:**

The spatial accuracy of the IFMM was found to be <0.1 mm. The measured latency was <200 ms. The dose difference with five interruptions was <0.5%.

**Conclusion:**

This work provides a quantitative characterization of the GKI IFMM system as required by the Nuclear Regulatory Commission. This provides a methodology for GKI users to satisfy these requirements using common laboratory equipment in lieu of a commercial solution.

## 1 INTRODUCTION

With the inception of the Gamma Knife Icon (GKI; Elekta Instrument AB, Stockholm Sweden), additional functionality has been added to the treatment system, including a cone beam computed tomography (CBCT) and an infrared camera‐based intra‐fraction motion management (IFMM) system allowing for frameless stereotactic radiosurgery. Additionally, new license guidance for use of the GKI in the United States^1^ has been released. The current license guidance from the Nuclear Regulatory Commission (NRC) for GKI dictates on a monthly basis the user will “confirm that the IFMM system is working properly by performing a test without a patient present with the aim to check the IFMM system's quantitative output.” Previous work has described commissioning a GKI system,^2^ the quality assurance, stability, and performance of the image guidance system,^3^, ^4^, ^5^ and described comparisons of the CBCT to IFMM;^6^, ^7^ however, the full quantitative characterization of the IFMM system is absent from all of these works. We are currently unaware of any commercial systems or published literature that allow the user to quantitatively test the temporal latency along with the spatial accuracy of the IFMM system as required by the NRC and as is recommended in current published radiation oncology quality assurance guidelines.^8^

The goal of this work was to quantitatively test and characterize the IFMM system. This includes the spatial accuracy of the IFMM, the ability of the IFMM to control the radiation unit of the Gamma Knife, the temporal latency of the system, and the temporal performance of the Gamma Knife sector drive unit. Furthermore, this work aims to make these quantifications safely from outside the vault during clinically realistic conditions to give the user confidence that the system will function as intended when treating patients.

## 2 MATERIAL AND METHODS

### 2.A Phantom construction

**Figure 1:**
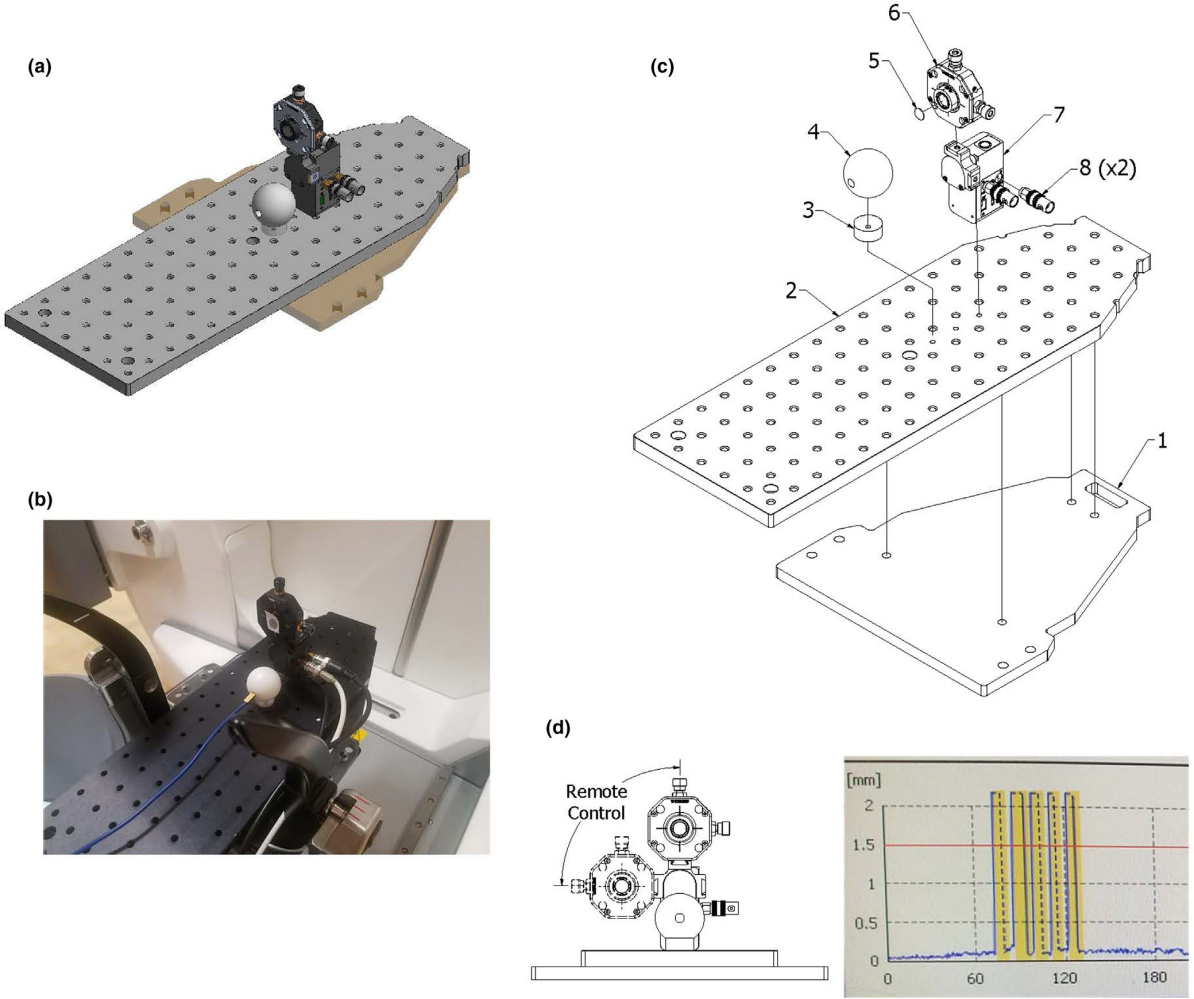
A computer‐aided design model of the constructed phantom is shown in (a). A picture of the phantom mounted on the treatment machine is shown in (b). (c) An exploded‐view drawing of the phantom consisting of (1) an acrylic plate, (2) optical breadboard, (3) a acrylic spacer, (4) thermoplastic sphere, (5) infrared marker, (6) translation stage, (7) flipper motor, and (8) SubMiniature version A to Bayonet Neill–Concelman adapters. (d) An example IFMM trace during a treatment showing the IFMM marker distance (blue points) on the Y axis as a function of time in seconds on the X axis. The five interruptions due to the phantom motion signaled by the user are shown in yellow.

Using computer aided design (CAD), a model of a phantom created and then constructed using common optical laboratory parts is shown in Fig. 1. The current mask adapter for GKI was used as a template for cutting a 10 mm thick acrylic base platform. Holes were drilled in the acrylic plate to match the mask registration pegs in the mask adapter [Fig. 1(b)]. The acrylic was cut to be flush with the outside of the mask adapter as clearance is limited between the CBCT arm and the mask adapter. Holes were then drilled to attach the acrylic plate to an optical breadboard. The optical breadboard was also cut to be flush with the side of the mask adapter and ground to avoid sharp edges. Two holes were then drilled in the breadboard near the middle of the mask adapter. This allowed for attachment of a remote motorized optical flipper (#MFF101, ThorLabs, Newton, NJ) to the optical breadboard in the most superior attachment location. A three‐axis translation optical mount (#CXYZ05, ThorLabs, Newton, NJ) was then attached to the remote motorized optical flipper. The remote flipper has two SubMiniature version A (SMA) coaxial RF connectors: (a) an input from the user's remote signal and (b) a 5 V transistor‐transistor logic (TTL) output channel. Using SMA to Bayonet Neill–Concelman adapters, the input and output channels of the flipper were attached via coaxial cable to the user outside of the vault. A 38 mm diameter thermoplastic acetal homopolymer resin sphere was tapped and drilled to be attached to the inferior attachment location on the breadboard (approximately 20 mm in front of the flipper and 70 mm lower than the marker). A 10 mm spacer was used to place the center of the sphere approximately 25 mm above the optical breadboard and close to the center of radiation unit focal point. The sphere was drilled with a 6.5 mm diameter bit for detector placement. An exploded‐view diagram of all these components can be seen in Fig. 1(c).

### 2.B Characterization and validation of IFMM

In Gamma Knife Leksell stereotactic space, the right posterior superior corner of the frame on a supine patient is (X = 0 mm, Y = 0 mm, Z = 0 mm) and the center of stereotactic space is (X = 100 mm, Y = 100 mm, Z = 100 mm), with XY being the axial plane, XZ being the coronal plane, and YZ being the sagittal plane. An infrared marker was placed at the center of the translation stage. The spatial accuracy of the IFMM was tested by moving each axis of the translation mount a known distance and recording the readout of the IFMM. Each axis has a calibrated micrometer screw that moves the stage a known amount per rotation, 250 μm per rotation of the screw in the X and Y directions, and 500 μm per rotation of the screw in the Z direction. The displacement according to the IFMM system is given as a magnitude on the treatment console. This value and its fluctuations were observed for each measurement and an average was taken.

**Figure 2:**
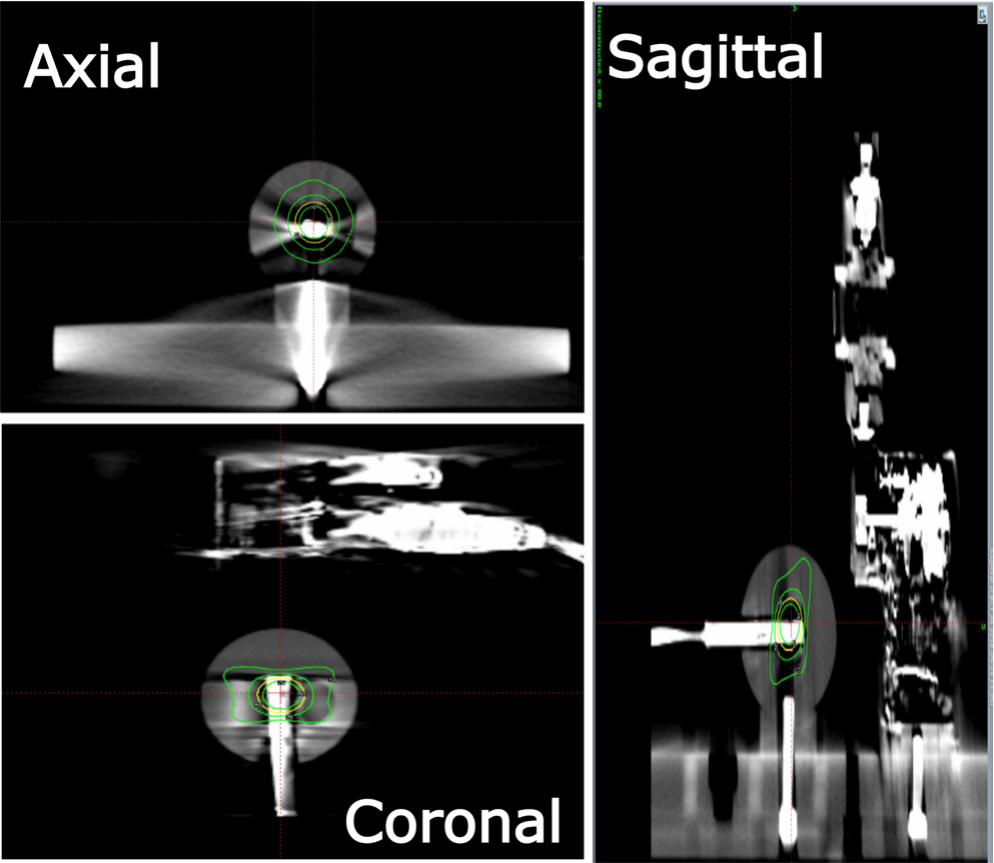
Axial, coronal, and sagittal views of an example treatment plan showing an 8 mm shot placed in the center of the detector volume.

Treatment plans were created post capturing a stereotactic reference CBCT of the phantom. Each plan was created to deliver a shot to the center of the detector located in the center of the thermoplastic sphere (X = 100.0, Y = 99.5, Z = 102.5). In the current version of the treatment planning software, the exterior skull definition cannot be completed using the CBCT images. The user must use a helical CT or magnetic resonance imaging (MRI) to define the skull. For this study, a skull was generated from a helical CT scan. Once completed, the plan was then approved, printed, and exported for treatment. Prior to treatment another CBCT was acquired to confirm the phantom position. To measure the latency of the IFMM system, the input of the optical flipper was connected to a remote outside of the treatment vault. The output of the flipper motor was connected to channel two of the dual channel data logging electrometer (PC Electrometer, Sun Nuclear, Melbourne, FL). A 0.01 mm^3^ (active volume) diode (Edge Detector, Sun Nuclear, Melbourne, FL) was inserted into the center of the thermoplastic sphere and connected to channel one of the electrometer. The current reading from each channel (channel 1 giving the edge detector signal and channel 2 giving the flipper motor output signal) was then simultaneously recorded vs time by the dual channel electrometer during the treatment of the plan. During the treatment delivery, the remote was pressed triggering the flipper to rotate 90° to a second fixed position [Fig. 1(d)]. The marker travels out of IFMM tolerance (=1.5 mm) in approximately 10 ms from triggering the motion. This is estimated given that the IR marker to the center of rotation distance is approximately 45 mm, and the arm rotates to 90° in 500 ms at a near constant velocity. The IFMM was used in the “Active” monitoring mode. In this mode, the sources move to the blocked sector position as soon as the IFMM threshold is exceeded and stay there unless the IR marker is back below threshold for at least 2 s. If the IFMM readout stays out of tolerance for more than 30 s, a treatment pause sequence is initiated by the system stopping the irradiation delivery and retracting both the sector and the patient couch to their corresponding home position and closing the treatment doors. Using the data from the electrometer one can see the time of trigger and the resultant beam of the Gamma Knife radiation unit as a function of time. The temporal difference of the two is the overall latency of the system. A sample IFMM trace is shown in Fig. 1(d). Since the shutter times for each collimator size on the Gamma Knife is different,^9^, ^10^, ^11^ this measurement was completed for all three shot sizes (4 mm, 8 mm, and 16 mm). The detector signals also show the time for sector movements from exposed to blocked positions. Axial, coronal, and sagittal views of an example treatment plan is shown in Fig. 2.

To test the overall dosimetric effect of these interruptions on the treatment, each shot was first delivered uninterrupted and the collected total charge was recorded. This was then compared to the total charge collected during irradiations with five sequential repeated interruptions approximately 10 s apart during the shot delivery. The ratio of these two readings was used to assess the effect of the interruptions on the treatment delivery. Five interruptions per shot is the maximum number of interruptions allowed without the unit initiating a treatment pause sequence. Each plan was created to deliver a constant dose to the center of the sphere, resulting in approximately a 1 min irradiation time for each shot.

## 3 RESULTS

### 3.A Spatial accuracy

Table 1 compares the IFMM reported displacements compared to translation stage displacements. Currently, the IFMM reports only the magnitude of displacement without direction. The real‐time fluctuations were within approximately ±0.05 mm of a given reported magnitude averaged over time by the observer. The maximum deviation from the micrometer to the IFMM was 0.05 mm with an average difference of 0.02 mm across all planes. Averaging the real‐time fluctuations of the IFMM output, the IFMM readings were found to be within 0.1 mm of the micrometer given all uncertainties. This test for spatial accuracy was completed at installation/full calibration of the Gamma Knife, and thus far has been reproducible during monthly spot check with three displacements (one in each plane) being tested every month.

**Table 1:** Translation stage displacements, IFMM reported displacements, and corresponding differences

Translation stage displacement (mm)	IFMM reported displacement magnitude (mm)	Difference between translation stage and IFMM (mm)
X = 0.25, 0.50, 0.75, 1.00, 1.25 X = −0.25, −0.50, −0.75, −1.00, −1.25	Mag = 0.26, 0.51, 0.77, 1.02,1.27 Mag = 0.27, 0.54, 0.78, 1.04, 1.28	ΔX = 0.01, 0.01, 0.02, 0.02, 0.02 ΔX = 0.02, 0.04, 0.03, 0.04, 0.03
Y = 0.25, 0.50, 0.75, 1.00,1.25 Y = −0.25, −0.50, −0.75, −1.00, −1.25	Mag = 0.27, 0.54, 0.78, 1.03, 1.29 Mag = 0.27, 0.53, 0.74,1.02,1.24	ΔY = 0.02, 0.04, 0.03, 0.03, 0.04 ΔY = 0.02, 0.03, −0.01, 0.02, −0.01
Z = 0.25, 0.50, 0.75, 1.00,1.25 Z = −0.25, −0.50, −0.75, −1.00, −1.25	Mag = 0.27, 0.54, 0.80, 1.04, 1.27 Mag = 0.23, 0.46, 0.79, 0.95, 1.26	ΔZ = 0.02, 0.04, 0.05, 0.04, 0.02 ΔZ = −0.02, −0.04, 0.04, −0.05, 0.01

### 3.B IFMM latency and temporal performance

**Figure 3:**
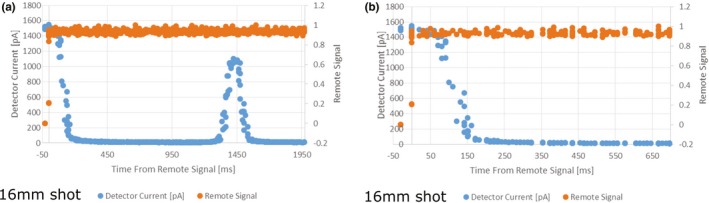
Detector current and remote trigger signal as a function of time during irradiation from a 16 mm shot. (a) is over the total time from the remote trigger to source being in the blocked position. (b) focuses on the time immediately before and after the remote trigger.

Data collected from the electrometer during the irradiation with the 16 mm collimator setting (Fig. 3) show the detector current as a function of time for the GK radiation unit transitioning from a beam on state to beam hold state when a trigger from the remote flipper motor is sent. For ease of analysis, the time of the trigger to the optical flipper was set to zero. One can see the total time for the sector to move from an exposed to a blocked state in Fig. 3. Due to the design of this generation of Gamma Knife, the source transits over the 4 mm collimator on the way to the blocked position from the exposed position of the 16 mm collimator.^11^ This can be seen at time, *t* = 1350 ms [Fig. 3(a)]. Traversing the 4 mm collimator takes approximately 200 ms. Focusing on the time immediately after the remote signal [Fig. 3(b)], at time = 60 ms the beam current has begun to drop and the sources are positioned between the 16 mm sector and the 4 mm sector 200 ms after the remote signal.

**Figure 4:**
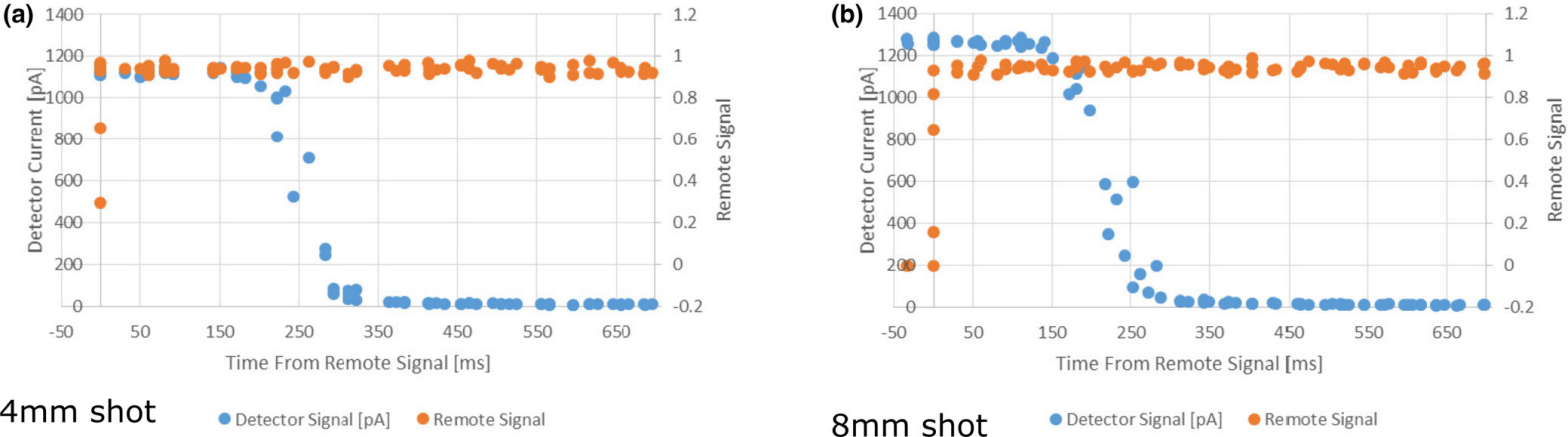
Detector current and remote trigger signal as a function of time during irradiation from a 4 mm shot (a) and an 8 mm shot (b).

Measurements were repeated with the 4 and 8 mm collimator settings. These collimator positions do not transit the sources over another collimator prior to going to the blocked position as the blocked position is between the 4 and 8 mm collimator position.^11^ Therefore, there is no second peak in detector signal post triggering the flipper (Fig. 4). One can see the time from the remote trigger to detector current decrease was 200 ms for the 4 mm collimator and 150 ms for the 8 mm collimator. The time for complete blocking of the sources was approximately 350 ms for both collimators [Figs. 4(a) and 4(b)].

The total charge collected for the uninterrupted shots was recorded for all three shot sizes. The same plans were then delivered while interrupting the shot five times. For the 16 mm collimator, the ratio of the interrupted reading over the uninterrupted reading was 103.2 nC/103.4 nC = 0.998. Similarly, for the 8 mm shot this ratio was 101.8 nC/102 nC = 0.998 and for the 4 mm shot this ratio was 99.04 nC/98.99 nC = 1.001.

## 4 DISCUSSION

Current license guidance^1^ and quality assurance guidelines^8^ mandate that the IFMM gating system should be quantitatively characterized. These properties include spatial accuracy, temporal accuracy, the ability to interlock the radiation beam, and the overall accuracy of the delivery. As this system is relatively new, to our knowledge, no commercial or vendor guidance is available for testing the system. This work describes a method that was developed to complete these tests using commercially available equipment, at a relatively low cost, in a radiation‐safe manner, and under clinically relevant conditions.

Using this method, tests indicate that the IFMM system performance, in terms of spatial accuracy (sub 0.1 mm), its ability to control the beam on/off states of the Gamma Knife radiation unit, and overall system latency (<200 ms), is capable for frameless stereotactic radiosurgery applications. Given that the dose rate of a 16 mm collimator at installation is approximately 3.5 Gy/min, a latency of 200 ms is clinically acceptable. With a 200 ms latency, the IFMM is equal to or faster than other clinical systems (optical marker or surface monitoring) currently being used for linear accelerator‐based radiosurgery and radiotherapy,^12^, ^13^ and cobalt‐based MRI‐guided radiotherapy systems.^14^

The IFMM system demonstrated an ability to control the radiation unit of the Gamma Knife reliably as the total dose delivered with and without interruption matched within 0.3%. Traversing the 4 mm collimator position when pausing or resuming a 16 mm sector was seen [Fig. 3(a)] and is a known consequence of the current generation Gamma Knife design. Due to shutter dose compensation at the treatment console^15^ and its relatively short exposure time (200 ms), this overall contribution of the interruption to the overall treatment dose, even with five interruptions in a given shot, is small (0.3% as we measured). This small error due to the interruptions is far outweighed by the benefit of the IFMM's functionality, that is, the IFMM detecting the patient moved and preventing dose being delivered to an area not accounted for in the treatment plan. Since the patient is not rigidly immobilized with a frame, the IFMM could potentially make the treatment very lengthy or even prevent the treatment all together if the patient is not compliant, thus highlighting the fact that patient selection is paramount for frameless SRS.

This work shows good agreement with previous works that showed a spatial accuracy of the IFMM to be 0.05 mm on average and within 0.16 mm maximally.^4^, ^5^ A limitation of this study is that measurements were performed on a single GKI unit. The performance of other GKI units may vary and would have to be characterized by an individual user following this methodology. Furthermore, these measurements were completed at the time of commissioning and periodically over a 6‐month period. At the time of writing the system performance is stable; however, there is no longer term data on the stability of the system's performance. While preliminary data suggest the system is stable, data will continue to be collected on a routine basis throughout the lifetime of the GKI at our institution to ensure this is true.

## 5 CONCLUSION

The IFMM system has been characterized and validated for use in frameless SRS on the GKI. The IFMM can achieve a spatial accuracy better than 0.1 mm and has system latency of less than 200 ms. Using the methodology presented here one can routinely test the IFMM system fulfilling requirements of the NRC with one phantom, safely from outside the treatment vault, giving the user confidence the system will function as intended when treating frameless radiosurgery patients.

## CONFLICT OF INTERESTS

No conflicts of interest.
